# The Mediating Effect of Changes in Depression Symptoms on the Relationship between Health-Related Quality of Life and Alcohol Consumption: Findings from a Longitudinal Study among Men Living with HIV in India

**DOI:** 10.3390/ijerph20085567

**Published:** 2023-04-18

**Authors:** Toan Ha, Hui Shi, Roman Shrestha, Sushma S Gaikwad, Kavita Joshi, Rupal Padiyar, Stephen L. Schensul

**Affiliations:** 1 Department of Infectious Diseases and Microbiology, School of Public Health, University of Pittsburgh, Pittsburgh, PA 15261, USA; 2 Department of Epidemiology, School of Public Health, University of Pittsburgh, Pittsburgh, PA 15261, USA; 3 Department of Allied Health Sciences, University of Connecticut, Storrs, CT 06269, USA; roman.shrestha@uconn.edu; 4 Medical College & B.Y.L. Nair Ch. Hospital, Mumbai 400008, India; 5 Seth G S Medical College, Mumbai 400012, India; 6 Lokmanya Tilak Municipal Medical College, Mumbai 400022, India; 7 Department of Public Health Sciences, University of Connecticut School of Medicine, Farmington, CT 06030, USA; schensul@uchc.edu

**Keywords:** HIV, health-related quality of life, depressive symptoms, alcohol use

## Abstract

Heavy alcohol use is negatively affecting antiretroviral therapy adherence, mental health and health-related quality of life among people living with HIV (PLWH). This paper aims to test the mediation model examining whether changes in depression symptoms mediate in the relationship between health-related quality of life and alcohol use among male PLWH who consume alcohol in India. The study is guided by the stress-coping model, which posits that individuals facing stress may turn to maladaptive coping mechanisms such as alcohol use to alleviate their distress, which includes depression and a low health-related quality of life due to various physical, psychological, and social factors associated with the HIV infection. This study used the data from a randomized controlled clinical trial entitled ‘Alcohol and ART adherence: Assessment, Intervention, and Modeling in India’. Participants completed surveys assessing demographic characteristics, health-related quality of life, depressive symptoms, and alcohol use. Multiple simple mediation models were investigated to examine whether changes in depression symptoms mediated the association between the changes in health-related quality of life and alcohol use after a 9-month follow-up. A total of 940 male PLWH were recruited and interviewed, with 564 participants in the intervention group and 376 participants in the control group. After a 9-month intervention, the mediation results showed that, among intervention participants, a decrease in depressiove symptoms mediated the relationship between improved health-related quality of life and lower alcohol use. However, among control participants, changes in depressive symptoms did not mediate the relationship between changes in health-related quality of life and alcohol use. The study findings have practical and theoretical implications. From a practical perspective, the results suggest that interventions aimed at simultaneously improving HRQoL and depressive symptoms among male PLWH with alcohol use may help reduce alcohol consumption. Therefore, interventions that address depressive symptoms in addition to improving HRQoL may have an even greater impact on reducing alcohol use among this population. Theoretically, the study supports the use of the stress-coping theory in understanding the association between HRQoL, mental health, and alcohol use among male PLWH, contributing to existing literature on a gap in our understanding of the interactions among these factors among PLWH.

## 1. Introduction

Human immunodeficiency virus (HIV) continues to remain a global public health concern despite significant advances in HIV prevention and treatment. With increasing access to effective HIV prevention, diagnosis, treatment and care, including for opportunistic infections, HIV has become a manageable chronic health condition, enabling people living with HIV (PLWH) to lead long and healthy lives [1]. This shift has also brought with it concerns about growing treatment demand as patients live longer, age-related chronic disease co-morbidities, and concerns about achieving social acceptance, stigma reduction, and psychological health [2]. It is, therefore, important to evaluate physical and emotional health among the changing demographics of PLWH. 

Quality of life refers to an individual’s subjective evaluation of their position in relation to their cultural and value systems, reference group, aspirations, expectations, standards, and concerns [3]. Health-related quality of life (HRQoL) specifically looks at how an individual’s health affects their ability to lead a satisfying life [4]. HRQoL reflects an individual’s perception and response to their health status and encompasses both health-related factors (e.g., physical, functional, emotional, mental well-being) and non-health-related factors (e.g., job, family, friends, life situations) [5]. As with other chronic conditions, PLWH face multiple challenges that can negatively impact various aspects of their lives [6]. PLWH, even when virologically suppressed, can still have a significantly lower HRQoL compared to the general population [7,8].

Likewise, one of the most prevalent psychiatric conditions among PLWH is depression symptoms, with rates ranging from 9% to 78%, particularly in low-and middle-income countries [9,10,11]. This represents a significant public health concern, as depression is a significant contributor to disability, accounting for nearly 7.5% of all disabilities [11]. Depression has also been found to be associated with negative psychological and health outcomes [12], including lower reported HRQoL, increased viral load, a faster progression to AIDS, and higher mortality rates among PLHW [12,13]. In addition, the rate of alcohol use disorder is continually rising, particularly among PLWH, and existing research has demonstrated adverse short-term and long-term impacts on physical, mental, and socio-economic well-being [14]. Specifically, studies from diverse geographic settings have shown higher levels of alcohol use associated with a reduction in HRQoL among PLWH [15], and higher levels of alcohol consumption have also been linked to increased depression symptoms among PLWH [16]. 

The association between alcohol use and HRQL has been also found not only in studies among PLWH, but also general populations [15,17,18]. Studies conducted among various populations have shown that those with higher levels of alcohol use had significantly lower HRQoL scores than those without alcohol use. For instance, heavy alcohol consumption was associated with a significant reduction in physical health, mental health, and overall quality of life scores in a cross-sectional study conducted among civil servants in China [18]. Conversely, a study in a national representative sample of adults in South Korea found that the HRQoL of those who consumed moderate alcohol was higher than that of non-drinkers and heavy drinkers [19]. However, the relationship between alcohol use and HRQoL is not one-directional, as evidenced by a 12-year longitudinal study involving 92,448 young and middle-aged women in the United States. The results showed that both moderate and heavy alcohol consumption were linked to lower HRQoL scores during the follow-up period. Furthermore, baseline poor HRQoL was associated with an increase in alcohol use over time, suggesting the bidirectional relationship between alcohol use and HRQoL and the complexity of this relationship [20]. 

It is estimated that there were 2.4 million PLWH in 2021 in India, making India the country with the third-highest number of PLWH in the world [21]. The prevalence of alcohol use is high among PLWH in India [22]. A recent study reported that 22.1% of male PLWH in India are considered habitual and social drinkers [23]. Additionally, previous studies among PLWH in India have found a high level of depression symptoms, ranging from 29% to 58.1% [17,24]. A recent cross-sectional study among men living with HIV who consume alcohol reported that nearly 40% of participants experienced moderate to high levels of depression symptoms [25]. 

Our study is based on the stress-coping model framework [26], which suggests that individuals who experience stress may resort to maladaptive coping mechanisms such as alcohol use to alleviate their distress. The stress-coping model has been widely used among various populations, including people living with HIV [27]. Positive coping strategies have been found to be associated with reduced levels of psychological distress, whereas negative coping strategies have been linked to increased levels of psychological distress [28]. One study among PLWH in the US showed that PLWH who report higher levels of negative coping strategies, such as self-blame, tend to have higher levels of alcohol use, while those who use positive coping strategies, such as seeking social support, exhibit lower levels of alcohol use [29]. The use of avoidance coping strategies, such as denial and substance use, has been associated with higher symptoms of depression and anxiety among PLWH initiating HIV care in Cameroon [30]. Additionally, the relationship between coping strategies and HRQOL has also been found to vary by gender among PLWH, with women reporting lower HRQOL scores and using negative coping strategies more frequently than men [31]. 

Based on the stress-coping theory, we propose that male PLWH may experience a decline in their HRQoL due to various physical, psychological, and social factors associated with the HIV infection. This decline in HRQL can lead to increased stress and negative emotions, including depression. As a result, some people with HIV may engage in maladaptive coping strategies, such as increased alcohol use, to manage these challenges. While informative, previous studies have mainly investigated the independent direct effects of HRQL, depression symptoms, and alcohol use on each other. However, there is still a gap in understanding how depression symptoms mediate the relationship between HRQoL and alcohol use in alcohol-consuming men living with HIV (PLWH) in low-resource settings such as India and other low- and middle-income countries. It is crucial to gain a better understanding of the extent of the indirect effect (i.e., mediation effect) to develop and implement targeted interventions. 

The purpose of this study was to assess the mediating role of depression symptoms in the relationship between HRQoL and alcohol use among male PLWH in India who consume alcohol. We tested the following hypotheses: improved HRQoL would be associated with reduced alcohol consumption; improvement in depression symptoms would be associated with reduced alcohol consumption; and improving HRQoL would lead to a decrease in depression symptoms, ultimately reducing alcohol consumption. The hypothesis was tested separately in both control and intervention groups. By comparing the outcomes between the two groups, we can assess the impact of the intervention on the relationship between HRQoL, depression symptoms, and alcohol use among male PLWH in India. Comparing results with a control group helps us draw stronger conclusions about the intervention’s effectiveness and control for other influencing factors.

## 2. Materials and Methods

### 2.1. Study Design and Sample Participants

This study utilized longitudinal data from the parent study, ‘Alcohol and ART Adherence: Assessment, Intervention, and Modeling in India’, which was a randomized controlled clinical trial designed to evaluate and compare the efficacy of a multi-level and multi-factorial intervention to decrease alcohol consumption and related factors among male PLHW on ART who consume alcohol in Mumbai, India [32,33]. The research was carried out in five randomly selected government ART centers from a total of 13 centers in Mumbai and Navi Mumbai, Maharashtra between 2014 and 2018. Eligibility criteria for study enrollment were: (1) on ART treatment for over 6 months; (2) had consumed alcohol during the last 30 days; and (3) 18 years of age and above. 

Study design: The study design employed an intervention crossover design, in which three types of interventions, including individual counseling (IC), group intervention (GI), and collective advocacy (CA), were implemented against two control ART centers, with the sequence of interventions varying by site. The ATR site 1 sequence was GI–IC–CA; the ATR site 2 sequence was IC–CA–GI; and the ATR site 3 sequence was CA–GI–IC. The intervention content focused on promoting healthy living, reducing depression, tension, and stress, reducing alcohol consumption, maintaining ART adherence, as well as promoting positive relationships and social support. A total of 940 male PLWH consented to participate in the study, with 564 participants receiving the three interventions and 376 participants in the two control ART centers. Both intervention and control participants continued to receive routine care approved by the India National AIDS Control Organization at the ART center while they were enrolled in the study. This study design allowed for the comparison of the effectiveness of the interventions with the standard care received by the control group. 

Baseline survey and follow-up evaluation: The pre-intervention baseline assessment was conducted at Time 1 (T1), followed by a follow-up assessment within 80 to 120 days after the intervention (T2). Of the 941 participants interviewed at T1, 871 had complete data on all study variables at both T1 and T2. The baseline and follow-up surveys were administered after medication pick-up to all participants in all five ART centers. The data collection was conducted orally in Hindi and Marathi by trained research investigators, and the data were recorded digitally on tablets using an Android-based app. 

### 2.2. Measurements

#### 2.2.1. Alcohol Use

The 10-item Alcohol Use Disorders Identification (AUDIT) scale, developed by the World Health Organization, was used to measure alcohol use [34]. The scale has 10 items and has been validated for use in India [35] (Cronbach’s alpha = 0.76). The responses for each item were scored from 0 to 4 with higher scores indicating a high level of alcohol use. Participants with a total score between 0–7 were classified as low risk, while those scoring from 8–16 were classified as hazardous risk [36].

#### 2.2.2. Health-Related Quality of Life (HRQoL)

The study used the Euro Quality of Life Five-Dimensions Three-Level (EQ-5D-3L) to assess HRQoL [37]. EQ-5D-3L consists of two parts: the EQ-5D descriptive system and the EQ visual analogue scale. The descriptive system evaluates five dimensions: mobility, self-care, usual activities, pain/discomfort, and depression/anxiety. Each dimension is rated on a three-point scale: 1 = no problem, 2 = moderate problem, 3 = severe problem. Participants were asked to rate their health in each dimension by choosing the most appropriate statement. The EQ-5D descriptive system was then converted into a single summary index score, ranging from 0 (worst/death) to 1 (perfect/full health), using a specific formula [38]. EQ-5D has been previously used and validated for use in the Indian population [39,40,41].

The EQ-5D-3L also includes a visual analog scale, which measures a participant’s self-rated overall health on a scale from 0 to 100. Participants were asked to mark a line indicating their current health state at the time of the interview, with 0 indicating the worst health and 100 representing the best.

#### 2.2.3. Depressive Symptoms

Depressive symptoms were measured by the 10-item Center for Epidemiologic Studies Depression (CESD) scale, which assesses various aspects of depression, including depressed mood, feelings of guilt, feelings of worthlessness, feelings of helplessness, loss of appetite, and sleep disturbance [42,43]. The CES-D has been validated for use in India [44]. The total score ranges from 0–30, with higher scores indicating increased symptom severity. The Cronbach coefficient alpha for this measure was 0.70.

#### 2.2.4. Demographic and Other Covariates

The demographic variables collected in the study included age, years of formal education, religion, household monthly income, and marital status. Other covariates included 4-day ART adherence, time since starting ART, time since HIV diagnosis, HIV-related stigma, family support, and HIV symptoms. ART adherence was measured using the AIDS Clinical Trials Group instrument, which elicited recall of missed doses over the four days prior to the survey. Scores below 100% indicated suboptimal adherence [45]. HIV-related stigma was assessed using the Berger 16-item HIV stigma scale [46], which consisted of four domains: disclosure concerns or anticipated stigma, negative self-image or internalized stigma, personalized stigma or enacted stigma, and concerns with public attitudes or anticipated stigma. Family support was evaluated using the 11-item medical outcomes study social support scale (α = 0.73) [23,47]. HIV symptoms were determined through self-reported symptoms related to HIV or ART medication [48]. Participants were asked about symptoms such as fatigue, dizziness, pain, nausea, diarrhea, sleep disturbance, skin problems, headache, loss of appetite, difficulty with sex satisfaction, body changes, weight loss, and hair loss experienced in the past 30 days.

### 2.3. Statistical Analysis

ANOVA and chi-square tests were employed to examine the difference of baseline characteristics by alcohol use risk (Low vs. Hazardous). Continuous variables were described as means and standard deviations while categorical variables were presented as frequency and percentages.

To investigate the mediation effect of changes in depression symptoms (measured at T2 compared to T1) on the relationship between changes in HRQoL (measured at T2 compared to T1) and alcohol use at T2, two simple mediation models were created using the mediation package in R (bruceR::PROCESS function, version 0.8.8) [49] to estimate the indirect effect, adjusting for various demographic and HIV-related factors such as age, education, marital status, religion, migration, household income, 4-day ART adherence, duration on ART (months), time since HIV diagnosis (months), HIV symptoms, family support, HIV-related stigma, and alcohol use risk at T1. The results were considered statistically significant if the *p*-value was less than 0.05. All analyses were performed using Stata statistical software version 17.0 (Stata Corp., College Station, TX, USA) and R statistical software version 4.0.3 (R Studio, R Foundation for Statistical Computing, Vienna, Austria). The mediation effect of changes in depression (T2-T1) on the effect of changes in HRQoL (T2-T1) and alcohol use at T2 was examined using a bias-corrected bootstrap method with 5000 simulations to calculate confidence intervals (Boot 95% CI). This method allows for the estimation of the indirect effect of depression on quality of life and alcohol use risk, through the use of bootstrapping to account for potential biases and uncertainty in the data. The results of this analysis can provide insight into the potential mechanisms by which changes in depression may influence changes in HRQoL and alcohol use. The coefficients will be considered statistically significant if the 95% CIs do not cross zero [50]. The proportion mediated (PM, %) was calculated by dividing the coefficient of the indirect effect by the coefficient of the total effect, as per the method proposed by Hayes. The results of the mediation analysis were reported in accordance with the Guideline for Reporting Mediation Analyses [51]. 

**Figure 1:**
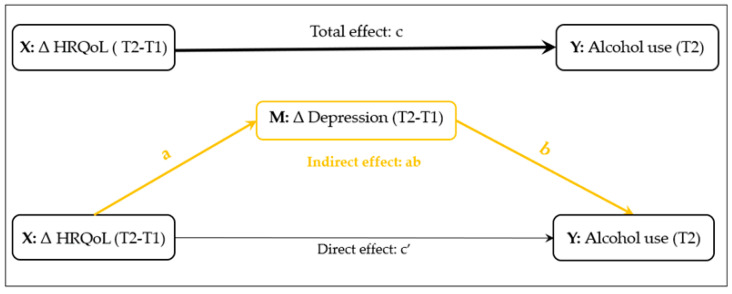
Conceptual diagram of the mediation model of change in depressive symptoms (ΔT2-T1) on the relationship between change in health-related quality of life (HRQoL) (ΔT2-T1) and alcohol use.

Mediation analysis. Multiple mediation models were conducted to investigate the impact of change in depression symptoms on the relationship between HRQoL and alcohol consumption, controlling for demographic and clinical variables. The mediation package in R software was used to estimate the coefficients of the total, indirect, and direct effects, and the bias-corrected bootstrap method was employed to calculate 95% confidence intervals for the coefficients. To investigate the mediation effects of change in depression on the relationship between HRQoL and alcohol consumption, we calculated the proportion of mediation (PM, %) using the indirect effect divided by the total effect. The mediation process is illustrated in Figure 1. The mediation hypotheses were tested using a three-step approach [52]. In step 1, we examined the total effect of HRQoL on alcohol use without considering the effect of depression symptoms. If there was a significant association, we moved on to step 2. In step 2, we examined the indirect effect of depression by analyzing the relationship between HRQoL and depression, as well as the relationship between depression and alcohol use. If both were significant, we moved on to step 3. In step 3, we examined the direct effect by including depression symptoms in the model from step 1. If the direct effect was not significant, it indicated that depression fully mediated the relationship between HRQoL and alcohol use. If it remained significant, it indicated partial mediation. We used a mediation package in R software and bias-corrected bootstrap to calculate 95% confidence intervals for the coefficients of the total, indirect, and direct effects [49]. *p*-value < 0.05 was considered statistically significant, and all analyses were performed using Stata Statistical Software (version 17.0, Stata Corp., College Station, TX, USA) and R Statistical Software (version 4.0.3.; R Foundation for Statistical Computing, Vienna, Austria).

## 3. Results

### 3.1. Characteristics of Participants

Table 1 and Table 2 show the baseline characteristics of intervention and control participants according to their alcohol use. Among intervention participants, those with lower ART adherence (*p* < 0.001), higher levels of depressive symptoms (*p* = 0.002), more family support (*p* = 0.001), and higher levels of HIV-related stigma (*p* = 0.017) were more likely to have greater alcohol use problems. Similarly, among control participants, those with lower ART adherence (*p* < 0.001), higher levels of depressive symptoms (*p* < 0.001), and higher levels of HIV-related stigma (*p* < 0.001) were more likely to engage in hazardous alcohol use.

### 3.2. Associations between Depression Symptoms and Other Factors

Table 3 presents the results of a multiple linear regression analysis investigating the factors affecting changes in depression symptoms from T1 to T2 among both intervention and control participants. The results reveal that in the intervention group, an improvement in quality of life was significantly correlated with a decrease in depressive symptoms (β = −0.111, 95% CI: −0.199 to −0.023, *p* = 0.013). In addition, baseline hazardous alcohol use was found to be associated with an increase in depressive symptoms (β = 0.131, 95% CI: 0.043 to 0.218, *p* = 0.003). In the control group, a significant association was observed between an increase in HRQoL and a decrease in depressive symptoms (β = −0.341, 95% CI: −0.445 to −0.237, *p* < 0.001). 

### 3.3. Associations between Alcohol Use and Other Factors

Table 4 presents the results of a multiple linear regression analysis examining factors affecting alcohol use at T2 among both intervention and control participants. The results indicate that, among the intervention participants, an improvement in HRQoL was linked to a reduction in alcohol use (β = −0.122, 95% CI: −0.204 to −0.040, *p* = 0.004), while an increase in depressive symptoms was associated with a higher risk of alcohol use (β = 0.174, 95% CI: 0.093 to 0.256, *p* < 0.001). However, there were no significant associations found between HRQoL, depressive symptoms, and alcohol use in the control group.

### 3.4. Mediating Effects

**Figure 2:**
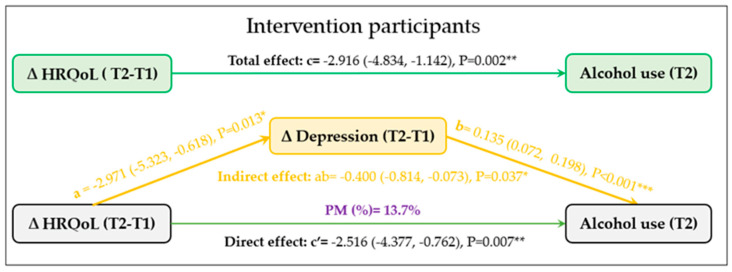
The mediation role of change in depressive symptoms (ΔT2-T1) on the relationship between change in health- related quality of life (HRQoL) (ΔT2-T1) and alcohol use (T2) among intervention participants (n = 519). * *p* < 0.05, ** *p* < 0.01, *** *p* < 0.001.

**Figure 3:**
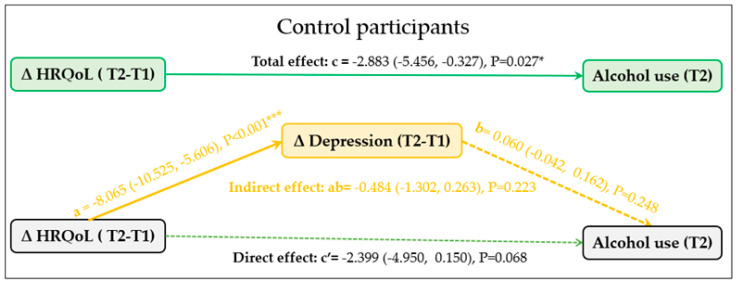
The mediation role of change in depressive symptoms (ΔT2-T1) on the relationship between change in health- related quality of life (HRQoL) (ΔT2-T1) and alcohol use (T2) among control participants (n = 352). * *p* < 0.05, *** *p* < 0.001.

The results of the mediation analysis, as shown in Figure 2, indicate that in the intervention group the impact of improved HRQoL on reduced alcohol consumption at T2 was partially mediated by reductions in depressive symptoms. The indirect effect of depression symptoms on the relationship between HRQoL and alcohol consumption was statistically significant, as evidenced by the Natural Indirect Effect (NIE) of −0.400 (95% CI: −0.814 to −0.073, *p* = 0.037). The proportion of the effect mediated by reductions in depressive symptoms was 13.7% of the total effect of HRQoL on alcohol use. In contrast, among control participants, a change in depressive symptoms did not mediate the relationship between change in HRQoL and alcohol use at T2 (Indirect effect: ab = −0.399, Boot 95% CI: −1.201 to 0.365, *p* = 0.314) (Figure 3).

## 4. Discussion

This study is one of the few that investigate the mediating role of depressive symptoms on the relationship between HQoL and alcohol use among male PLWH who consume alcohol. Our findings support the hypothesis that a reduction in depressive symptoms mediates the effect of improvement in HRQoL on a reduction of alcohol use. Specifically, the results show that improvements in HRQoL in the intervention arm resulted in a reduction in depression, which in turn led to decreased alcohol consumption. This effect was not observed in the control arm. The intervention arm of this study involved a multilevel approach to address several factors, including alcohol use, depressive symptoms, healthy living, and ART adherence. This was achieved through individual counseling, group interventions, and collective action. Both individual counseling and group interventions included a significant component that addressed depressive symptoms, stress, healthy living, and relationships as well as an alcohol risk-reduction module. The collective action intervention focused on identifying key structural problems affecting PLWH and planning and conducting action to address structural changes. In contrast, the control group received only standard care from the ART center, without the additional multilevel interventions provided to the intervention group. The study’s results have significant public health implications and suggest that reducing alcohol use among male PLWH who consume alcohol may require a combination of interventions at different levels that address HRQoL and depressive symptoms as well as other factors concurrently.

### 4.1. HRQoL and Alcohol Use

Our findings also indicated that changes in HRQoL had a direct effect on alcohol use, with an improvement in HRQoL being associated with a reduction in alcohol consumption. Based on the stress-coping theory, the direct link between HRQoL and alcohol use may be explained by the fact that a person’s HIV status can result in a decline in HRQoL as they go through familial, psychological social and mortality risk concerns associated with the HIV infection. Those with low HRQoL may turn to alcohol to cope with the difficulties associated with their conditions. Results from our prior studies among this population indicated that participants drank to cope with emotions such as sadness or physical discomfort due to their HIV symptoms. Previous studies have also reported that the presence of HIV-related symptoms is associated with a poor quality of life [53]. The findings of our study suggest that improving HRQoL will be an important factor in reducing alcohol consumption among PLWH. However, the relationship between alcohol use and HRQoL is more complex and not one-directional, as indicated by a 12-year longitudinal study involving 92,448 young and middle-aged women in the United States. The results showed that both moderate and heavy alcohol consumption were linked to lower HRQoL scores during the 12-year follow-up period. Interestingly, baseline poor HRQoL was also associated with an increase in alcohol use over time, highlighting the bidirectional nature of this relationship [20]. This suggests the need for further study on the relationship between HRQoL and alcohol use among various populations. 

### 4.2. Depression Symptoms and Alcohol Use

This study has shown that higher levels of depressive symptoms were directly related to increased alcohol use among PLWH. These results are consistent with previous studies [54], which found that higher levels of depressive symptoms were associated with increased alcohol consumption among PLWH [16]. Previous studies suggest that some PLWH may use alcohol as a way to cope with emotional difficulties, such as depression [55]. 

This study had several strengths, including the collection of longitudinal data that allows for an evaluation of causality over time. It employed a randomized controlled trial design, which is considered the most stringent method for evaluating treatment outcomes. Furthermore, the large sample size improves the representativeness of the results, and these findings have great importance in advancing our understanding of the complex relationship between HRQoL, depressive symptoms, and alcohol use.

### 4.3. Limitations

This study has several limitations. The study population consists of only male PLWH who consume alcohol from five ART centers, which limits the generalizability of the results to other non-drinking and diverse populations affected by HIV. The results are based on self-reported data, which can be prone to biases and social desirability effects. Additionally, while our analyses took into account major confounding factors, it is possible that residual confounding still exists. Therefore, our findings and quantitative estimates should be interpreted with caution.

## 5. Conclusions

The study, utilizing longitudinal data, found that depression symptoms acted as a mediator in the relationship between HRQoL and alcohol use among male PLWH who consume alcohol. The findings of this study have practical and theoretical implications. From a practical perspective, the results suggest that interventions aimed at improving simultaneously HRQoL and depression symptoms among male PLWH with alcohol use may help reduce alcohol consumption. Therefore, interventions that address depressive symptoms in addition to improving HRQoL may have an even greater impact on reducing alcohol use among this population. Theoretically, the study supports the use of the stress-coping theory in understanding the association between HRQoL, mental health, and alcohol use among male PLWH, contributing to existing literature on a gap in our understanding of the interactions among these factors among PLWH. 

## Figures and Tables

**Table 1:** Sample characteristics at T1 among intervention participants (n = 564).

		Alcohol Use		
Characteristics	Overall, n = 564	Low, n = 354	Hazardous, n = 210	*p*-Value
**Age** (in years), mean (range)	42.0 (19.0–75.0)	42.0 (19.0–72.0)	42.0 (22.0–75.0)	0.831
**Education background**, *n* (%)				0.166
Illiterate	62.0 (11.0%)	31.0 (8.8%)	31.0 (14.8%)	
Primary school	134.0 (23.8%)	87.0 (24.6%)	47.0 (22.4%)	
Secondary school	343.0 (60.8%)	221.0 (62.4%)	122.0 (58.1%)	
High school and above	25.0 (4.4%)	15.0 (4.2%)	10.0 (4.8%)	
**Marital status**, *n* (%)				0.837
Currently married	463.0 (82.1%)	293.0 (82.8%)	170.0 (81.0%)	
Never married	49.0 (8.7%)	29.0 (8.2%)	20.0 (9.5%)	
Separated/divorced/widowed	52.0 (9.2%)	32.0 (9.0%)	20.0 (9.5%)	
**Religion**, *n* (%)				0.211
Hindu	491.0 (87.1%)	313.0 (88.4%)	178.0 (84.8%)	
Other	73.0 (12.9%)	41.0 (11.6%)	32.0 (15.2%)	
**Migration**, *n* (%)				0.386
Born in Mumbai	354.0 (62.8%)	227.0 (64.1%)	127.0 (60.5%)	
Born outside Mumbai	210.0 (37.2%)	127.0 (35.9%)	83.0 (39.5%)	
**Household income per month**, *n* (%)				0.819
<20,000 INR	454.0 (80.5%)	286.0 (80.8%)	168.0 (80.0%)	
≥20,000 INR	110.0 (19.5%)	68.0 (19.2%)	42.0 (20.0%)	
**4-day ART adherence**, *n* (%)				<0.001 *
≥90%	453.0 (80.3%)	300.0 (84.7%)	153.0 (72.9%)	
<90%	111.0 (19.7%)	54.0 (15.3%)	57.0 (27.1%)	
**Duration on ART (months)**, mean (SD)	51.5 (32.8)	51.6 (33.4)	51.2 (31.8)	0.952
**CD4 count (cells/mL)**, mean (SD)	469.6 (239.3)	469.3 (235.0)	470.0 (246.8)	0.894
**HIV symptoms**, mean (SD)	3.4 (3.0)	3.1 (2.9)	3.8 (2.9)	0.002 *
**Family support**, mean (SD)	5.1 (3.0)	4.7 (3.1)	5.6 (2.8)	0.001 *
**HIV-related stigma**, mean (SD)	42.6 (7.4)	42.2 (7.5)	43.3 (7.0)	0.017 *

Abbreviations: SD, standard deviation; *ART*, antiretroviral treatment; *CESD*, 10-item Center for Epidemiologic Studies Depression Scale; * *p* < 0.05.

**Table 2:** Sample characteristics stratified by alcohol use at T1 among control participants (n = 376).

		Alcohol Use Risk		
Characteristics	Overall, n = 376	Low, n = 295	Hazardous, n = 81	*p*-Value
**Age** (in years), mean (range)	44.3 (19.0–71.0)	44.3 (19.0–71.0)	44.2 (26.0–64.0)	0.988
**Education background**, *n* (%)				0.994
Illiterate	25.0 (6.6%)	20.0 (6.8%)	5.0 (6.2%)	
Primary school	75.0 (19.9%)	58.0 (19.7%)	17.0 (21.0%)	
Secondary school	260.0 (69.1%)	204.0 (69.2%)	56.0 (69.1%)	
High school and above	16.0 (4.3%)	13.0 (4.4%)	3.0 (3.7%)	
**Marital status**, *n* (%)				0.402
Currently married	274.0 (72.9%)	219.0 (74.2%)	55.0 (67.9%)	
Never married	53.0 (14.1%)	41.0 (13.9%)	12.0 (14.8%)	
Separated/divorced/widowed	49.0 (13.0%)	35.0 (11.9%)	14.0 (17.3%)	
**Religion**, *n* (%)				0.574
Hindu	336.0 (89.4%)	265.0 (89.8%)	71.0 (87.7%)	
Other	40.0 (10.6%)	30.0 (10.2%)	10.0 (12.3%)	
**Migration**, *n* (%)				0.240
Born in Mumbai	221.0 (58.8%)	178.0 (60.3%)	43.0 (53.1%)	
Born outside Mumbai	155.0 (41.2%)	117.0 (39.7%)	38.0 (46.9%)	
**Household income per month**, *n* (%)				0.331
<20,000 INR	315.0 (83.8%)	250.0 (84.7%)	65.0 (80.2%)	
≥20,000 INR	61.0 (16.2%)	45.0 (15.3%)	16.0 (19.8%)	
**4-day ART adherence**, *n* (%)				<0.001 *
≥90%	336.0 (89.4%)	272.0 (92.2%)	64.0 (79.0%)	
<90%	40.0 (10.6%)	23.0 (7.8%)	17.0 (21.0%)	
**Duration on ART** (in months)*,* mean (SD)	57.9 (29.7)	58.8 (29.4)	54.6 (30.7)	0.291
**CD4 count** (cells/mL), mean (SD)	467.1 (215.0)	468.6 (218.1)	461.4 (204.9)	0.983
**HIV symptoms***,* mean (SD)	2.4 (2.2)	2.2 (2.3)	3.2 (1.9)	<0.001 *
**Family support***,* mean (SD)	2.9 (2.9)	2.8 (2.9)	3.3 (2.6)	0.102
**HIV-related stigma***,* mean (SD)	36.9 (5.5)	36.4 (5.3)	39.1 (5.7)	<0.001 *

Abbreviations: SD, standard deviation; ART, antiretroviral treatment; *CES-D*, a 10-item Center for Epidemiologic Studies Depression Scale. * *p* < 0.05.

**Table 3:** Multiple linear regression of factors associated with changes in depression symptoms (T2-T1).

	Intervention Participants (n = 519) ^¶^		Control Participants (n = 352) ^⸸^	
Characteristics	β (95% CI)	*p*-Value	β (95% CI)	*p*-Value
**Age** (in years)	−0.073 (−0.163, 0.018)	0.115	−0.039 (−0.145, 0.067)	0.466
**Education background** (ref: Illiterate)				
Primary school	0.040 (−0.068, 0.148)	0.469	0.083 (−0.044, 0.210)	0.200
Secondary school	−0.039 (−0.133, 0.055)	0.415	0.001 (−0.104, 0.107)	0.979
High school and above	0.003 (−0.096, 0.102)	0.952	0.058 (−0.061, 0.177)	0.340
**Marital status** (ref: Never married)				
Currently married	0.051 (−0.044, 0.147)	0.293	−0.084 (−0.192, 0.024)	0.128
Separated/divorced/widowed	−0.004 (−0.100, 0.092)	0.934	−0.059 (−0.172, 0.053)	0.299
**Religion** (ref: Others)				
Hindu	−0.042 (−0.129, 0.044)	0.337	0.001 (−0.101, 0.098)	0.103
**Migration** (ref: Born outside Mumbai)				
Born in Mumbai	0.046 (−0.044, 0.136)	0.313	0.0530(−0.057, 0.163)	0.348
**Household income per month** (ref: <20,000 INR)				
≥20,000 INR	0.029 (−0.058, 0.116)	0.509	−0.030 (−0.133, 0.074)	0.577
**4-day ART adherence** (ref: <90%)				
≥90%	0.059 (−0.030, 0.147)	0.193	−0.073 (−0.176, 0.030)	0.165
**Duration on ART** (months)	0.055 (−0.035, 0.145)	0.231	0.080 (−0.022, 0.181)	0.124
**CD4 count** (cells/mL)	−0.085 (−0.172, 0.002)	0.056	−0.112 (−0.214, 0.011)	0.030
**HIV symptoms**	−0.115 (−0.203, −0.027)	0.011 *	−0.008 (−0.120, 0.104)	0.889
**Family support**	−0.017 (−0.106, 0.072)	0.712	0.070 (−0.043, 0.183)	0.222
**HIV-related stigma**	0.024 (−0.063, 0.111)	0.583	−0.113 (−0.220, −0.006)	0.039 *
**Alcohol use at T1** (ref: Low risk)				
Hazardous	0.131 (0.043, 0.218)	0.003 *	−0.066 (−0.168, 0.036)	0.205
**HRQoL Δ from T1-T2**	−0.111 (−0.199, −0.023)	0.013 *	−0.341 (−0.445, −0.237)	<0.001 *

Abbreviations: depressive symptoms Δ = Change in depressive symptoms since baseline (T1); HRQoL Δ = HRQoL changes since baseline (T1); ^¶^ Model Fit: F (17, 501) = 2.42, *p* = 0.001, R² = 0.076 (Adjusted R² = 0.044); ^⸸^ Model Fit: F (17, 334) = 4.07, *p* < 0.001, R² = 0.172 (Adjusted R² = 0.130). * *p* < 0.05.

**Table 4:** Multiple linear regression of factors associated with alcohol use at T2.

	Intervention Participants (n = 519) ^¶^		Control Participants (n = 352) ^⸸^	
Characteristics	β (95% CI)	*p*-Value	β (95% CI)	*p*-Value
**Age** (in years)	−0.053 (−0.137, 0.032)	0.221	−0.080 (−0.186, 0.027)	0.141
**Education background** (ref: Illiterate)				
Primary school	−0.065 (−0.162, 0.035)	0.201	0.051 (−0.076, 0.178)	0.432
Secondary school	0.004 (−0.083, 0.091)	0.936	−0.081 (−0.178, 0.024)	0.130
High school and above	−0.064 (−0.156, 0.027)	0.168	−0.004 (−0.123, 0.115)	0.945
**Marital status** (ref: Never married)				
Currently married	0.033 (−0.055, 0.122)	0.460	0.005 (−0.103, 0.114)	0.924
Separated/divorced/widowed	−0.039 (−0.128, 0.050)	0.388	0.052 (−0.061, 0.164)	0.368
**Religion** (ref: Others)				
Hindu	−0.067 (−0.148, 0.013)	0.101	0.085 (−0.017, 0.187)	0.101
**Migration** (ref: Born outside Mumbai)				
Born in Mumbai	0.172 (0.089, 0.256)	<0.001 *	−0.001 (−0.110, 0.110)	0.997
**Household income per month** (ref: <20,000 INR)				
≥20,000 INR	0.063 (−0.018, 0.143)	0.127	0.005 (−0.099, 0.109)	0.923
**4-day ART adherence** (ref: <90%)				
≥90%	−0.005 (−0.087, 0.077)	0.909	−0.038 (−0.141, 0.065)	0.464
**Duration on ART** (months)	0.026 (−0.057, 0.110)	0.540	0.091 (−0.011, 0.193)	0.080
**CD4 count** (cells/mL)	0.012 (−0.069, 0.092)	0.779	−0.028 (−0.074, 0.131)	0.585
**HIV symptoms**	−0.010 (−0.093, 0.072)	0.805	−0.161 (−0.273, −0.049)	0.005 *
**Family support**	−0.069 (−0.151, 0.014)	0.102	−0.047 (−0.160, 0.066)	0.415
**HIV-related stigma**	0.083 (0.002, 0.164)	0.044	−0.060 (−0.167, 0.048)	0.276
**Alcohol use at T1** (ref: Low risk)				
Hazardous	0.253 (0.172, 0.335)	<0.001 *	0.330 (0.227, 0.432)	<0.001 *
**HRQoL Δ from T1-T2**	−0.122 (−0.204, −0.040)	0.004 *	−0.107 (−0.217, 0.003)	0.057
**Depression Δ from T1-T2**	0.174 (0.093, 0.256)	<0.001 *	0.063 (−0.044, 0.171)	0.248

Abbreviations: Depressive symptoms Δ = change in depressive symptoms since baseline (T1); HRQoL Δ = HRQoL changes since baseline (T1). ^¶^ Model Fit: F (18, 500) = 7.36, *p* <0.001, R² = 0.210 (Adjusted R² = 0.181); ^⸸^ Model Fit: F (18, 333) = 3.92, *p* < 0.001, R² = 0.175 (Adjusted R² = 0.130); * *p* < 0.05.

## Data Availability

The data presented in this study are available on request from the corresponding author.
